# Gut microbiota diversity affects fish behaviour and is influenced by host genetics and early rearing conditions

**DOI:** 10.1098/rsob.240232

**Published:** 2025-04-16

**Authors:** Ishrat Z. Anka, Tamsyn Uren Webster, Sam McLaughlin, Benjamin Overland, Matthew Hitchings, Carlos Garcia de Leaniz, Sofia Consuegra

**Affiliations:** ^1^ Department of Biosciences, Swansea University, Swansea, UK; ^2^ Department of Aquaculture, Chattogram Veterinary and Animal Sciences University, Chittagong, Bangladesh; ^3^ Institute of Life Sciences, Swansea University, Swansea, UK; ^4^ Centro de Investigaciones Marinas, Universidade de Vigo, Vigo, Spain; ^5^ Departamento de Biotecnologia y Acuicultura, Instituto de Investigaciones Marinas (IIM-CSIC), Vigo, Spain

**Keywords:** host–microbiome interaction, probiotic, environmental enrichment, fish microbiome, novel object

## Abstract

The gut microbiota influences human and animal cognition and behaviour through its effects on the endocrine and immune systems. The microbiome–behaviour relationship may be especially relevant for fish, due to their diverse evolutionary history and potential implications for farming and conservation. Yet, there is limited research on the interaction between gut microbiome and behaviour in non-model fish. We manipulated the rearing environment and diet of fish from two inbred strains of the self-fertilizing mangrove killifish (*Kryptolebias marmoratus*) and assessed the effects on the gut microbiome and its interactions with anxiety-like behaviours. We found that microbiota composition and alpha diversity were significantly influenced by host genetics (strain), hatching mode (naturally or artificial dechorionation) and diet, but not by environmental enrichment. Fish activity level and inspections of a novel object were strongly associated with microbiota community composition and alpha diversity. The microbial taxa associated with differences in behaviour were dominated by Bacteroidales, potentially related to the production of metabolites affecting neural development. We suggest that the association between microbiome and fish behaviour could be an indirect effect of the modulation of the gut microbiota by host genetics and early rearing conditions, which could be affecting the production of microbial metabolites that interact with the fish physiology.

**Figure:**
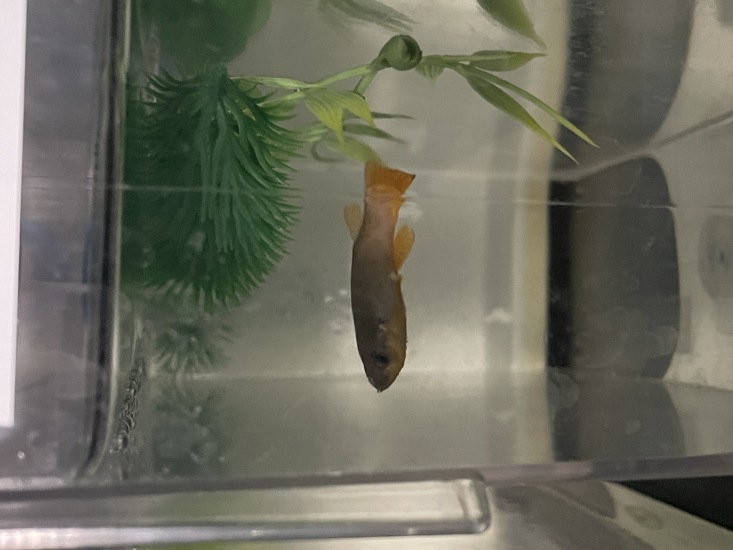

## 1. Background

The gut microbiota influences human and animal cognition and behaviour [1,2] through its effects on the endocrine and immune systems [3]. This knowledge is opening novel avenues for research, particularly for the treatment of neurodevelopmental diseases [4], although the mechanisms and evolutionary significance of the microbiome–behaviour relationship are still unclear [5]. Until recently, genetics and the environment were considered the main factors influencing the development of the brain. However, human correlational analyses and experimental studies with germ-free mice indicate that the early colonizing microbiota are also involved in neurodevelopmental processes, by initiating signalling mechanisms that affect neuronal circuits involved in motor control and anxiety-like behaviours [6], and their disruption can have long-lasting effects on brain function [7]. In mammals, pathological stress or disease can lead to intestinal dysbiosis which negatively influences gut physiology, altering the brain–gut axis signalling and, as a consequence, the central nervous system functioning [4].

While the association between neurodevelopment and microbiome has long been considered in the fields of medicine and psychology [8], its relevance for animal applications such as captive breeding for commercial or conservation purposes remains largely unexplored [9]. Yet, understanding the role of the microbiome in mediating behaviour and social interactions in this context is particularly relevant for the welfare and long-term survival of animal populations. For example, in non-human primates, analyses of the gut microbiome have indicated that social grouping and physical interactions are the most important drivers of the gut microbiome composition, maintaining commensal and mutualistic microbial diversity which provides disease protection across generations [10]. The microbiome–behaviour relationship may be highly relevant for fish, due to their high diversity, variety of habitats and complex social behaviours [11], but the number of studies is still very limited. Zebrafish studies suggest that, as in mammals, early life microbiota is critical for normal neural development [12]. Germ-free zebrafish and larvae supplemented with the probiotic *Lactobacillus plantarum* display less anxiety-related behaviours compared with controls raised under standard conditions, supporting the suggestion that the microbiome plays a role in regulating the stress response in this fish [13]. Skin microbiota also seems to influence activity levels in fish in a sex-specific manner, as a recent study in guppies indicated [14]. The association between microbiome and behaviour can be particularly important given the negative effects that stress can have in the fish skin and gut microbiomes [15].

The relationship between the host genetic background, environmental factors and the microbiota, and their joint effect on the host cognitive behaviour, merits particular attention [1]. Host genetics influences microbial composition and ecology, while the gut microbiota influences the host neurological function through the gut–brain axis, making it critical to disentangle the role of these reciprocal interactions in the host behaviour, which may result from their synergistic effects [16]. For example, the maladaptive behaviour of Cntnap2^−^*^I−^* mice, which are models for neurodevelopmental disorders, results from a combination of abnormal social behaviour mediated by the gut microbiome and hyperactive locomotor activity level controlled by a genetic mutation [16]. Co-varying factors such as genetic variation, age, environmental conditions, diet or medication are common confounding factors in the analyses of the association between the microbiome and neurological development or social behaviour [17]. Germ-free organisms reared in controlled laboratory conditions are often used to avoid some of this experimental co-variation [18]. Yet, while the use of germ-free animals can give important information for cognitive diseases and pathological behaviours, doing manipulative experiments of the actual microbiome (using probiotics, prebiotics or animal translocations, for example [19]) is a more powerful approach to study the implications of changes of the microbiota in normal animal behaviours [20].

Environmental enrichment is often used in aquaculture and laboratory conditions to decrease fish stress [21], promote more natural behaviours [22–24] or improve foraging performance [25]. Dietary supplements (prebiotics and probiotics) are also frequently applied in aquaculture and laboratory fish feeds, to enhance fish health (immnunocompetence) [26], reduce stress [27,28] and increase welfare [29,30]. Here, we used a self-fertilizing species, the mangrove killifish (*Kryptolebias marmoratus*), to assess the relative roles of the microbiome, host genetics and early rearing environment on fish behaviour. The self-fertilizing nature of the study species, with naturally inbred and genetically homogeneous strains, makes it ideal to disentangle genetic and environmental effects [31]. We manipulated the environment (using environmental enrichment) and the microbiome (incorporating a probiotic in the diet) and assessed the exploratory behaviour and activity level of two distinct killifish strains using a novel object experimental approach, previously used in this species to identify behavioural variation [31].

## 2. Methods

### 2.1. Experimental setting

Two genetically different strains of the naturally inbred mangrove killifish *K. marmoratus* (DAN and HON9) were used to assess the relationship between microbiome and behaviour in response to changes in the rearing environment. These lines have different geographic origins (Belize for DAN and Honduras for HON9) [32] and have undergone >30 generations of inbreeding under laboratory conditions.

Eggs were collected daily from 10 self-fertilizing adult *K. marmoratus* (five from each line) and individually kept in 50 ml of brackish water under standard conditions (12:12 h light/darkness, temperature 20–26°C, approx. 14 ppt salinity) until hatching, being monitored on a regular basis to check developmental stages throughout the incubation period. Hatching took place either naturally (DAN *n* = 16 and HON9 *n* = 36) or through artificial dechorionation after approximately 30 days if they had not hatched naturally (for DAN *n* = 24 and for HON9 *n* = 4). After hatching, individual alevins (*n* = 80, 40 from each strain) were reared individually in rectangular plastic tanks (16.5 cm long × 11 cm wide × 10 cm high) with 500 ml of brackish water at 14 ppm salinity approximately, under one of two physical enrichment conditions (enriched and poor) and fed one of two possible diets (artemia enriched with Easy Dry Seclo (EDS) or enriched with a probiotic). The probiotic enrichment consisted of a combination of three *Bacillus* species (*Bacillus subtilis*, *B. licheniformis* and *B. pumilus*; 1 × 10^10^ cfu g^−1^), formulated for fish farming. The enrichment of the diet with probiotic followed the protocol in [33] (see details in electronic supplementary material, Methodology). Enriched environment tanks contained one artificial plastic log (approx. 5 cm long and approx. 3.5 inch diameter) and two artificial small plants which the poor environment tanks lacked [31]. In total, there were four experimental groups (combination of two physical enrichment and two diet conditions) with 10 fish per group for each genotype (electronic supplementary material, table S1, figure S1). Forty-six of these fish (DAN *n* = 26, HON9 *n* = 20) were analysed for both microbiome and behaviour.

### 2.2. Behavioural tests

A novel object exploration test was performed on individual fish approximately 10 months old as in [31]. Fish were not fed for 24 h prior to the experiment, and were then transferred from the rearing tank to a custom-made experimental tank (electronic supplementary material, figure S2) and acclimated for approximately 15 min in an isolated area. Tanks were divided into 5 zones, with 0 being the acclimation zone and 5 the furthest from it, the novel object (a Lego piece) was located in the middle of the tank (Zone 3). Individual fish behaviour was recorded for 20 min using overhead cameras. All recorded videos were analysed using BORIS v. 7.12.2 [34] and the following behaviours were recorded: (i) exploration: proportion of time spent in all exploratory zones (Zones 1−5) throughout the experiment duration (20 min); (ii) inspections: the number of times the fish inspected the novel object zone (Zone 3); (iii) activity: total number of crosses among zones (Zones 0−5); and (iv) contacts: number of contacts with the novel object. The last measurement was subsequently removed from analyses due to the very low number of contacts, which was less than one on average.

### 2.3. DNA extraction and library construction

Individual killifish from each experimental group were euthanized following Schedule 1 of the Animals Act, using a solution of 2-phenoxyethanol (1 ml l^−1^). Individual whole gut samples were preserved in RNAlater at −80℃. DNA extraction was performed using the DNeasy PowerLyzer PowerSoil Kit (QIAGEN) [35]. Amplification of the 16S rRNA gene-V4 region [36] was performed using primers 515F:GTGCCAGCMGCCGCGGTAA [37] and 806R:GGACTACHVGGGTWTCTAAT [38]. PCR_1 consisted of a total volume of 22.5 µl incorporating 12.5 µl of Platinum™ II Hot-Start PCR Master Mix (2×) (Thermo Fisher Scientific), 0.5 µl of forward (FP) and reverse (RP) primers (10 µM), 9 µl of ultra-pure water (UPW) and 2.5 µl of DNA. The PCR began with a 3 min denaturation step at 95℃ followed by 27 cycles of 95℃ for 30 s, 55℃ for 30 s and 72℃ for 30 s, then a final elongation step at 72°C for 5 min. During PCR_2, indexing with Nextera^®^ XT Index Kit v2 (Illumina, Inc., San Diego, CA, USA) was performed. PCR_2 was made with a total volume of 27.5 µl per sample, containing 2.5 µl of PCR_1 product, 1.25 µl of each index, 12.5 µl of Platinum™ II Hot-Start PCR Master Mix (2×) and 10 µl of UPW. The reaction conditions were as above and with 11 cycles for PCR_2. Final PCR products were pooled from samples (*n* = 46) based on agarose gel band intensity and cleaned using AMPure XP beads (Beckman Coulter Genomics, Brea, CA, USA). Final library quantification was performed using qPCR (NEB Illumina quantification kit), prior to sequencing on a MiSeq Illumina platform (300 bp, paired end). A blank was extracted and sequenced alongside the samples and yielded 990 reads that were reduced to 20 reads after quality filtering.

### 2.4. Bioinformatics analysis

Sequence data were processed in Qiime2 (version: qiime2-2022.2) [39]. DADA2 [40] was used to assemble amplicon sequence variants (ASVs), after truncating forward (220 bp) and reverse (155 bp) reads based on the quality scores. ASVs were classified using the Silva reference taxonomy (v138) [41], after which mitochondrial, chloroplast and unclassified reads were removed. Sub-sampling was carried out to a total of 9906 reads for each sample (*n* = 46). ASVs were further filtered to retain only those present in at least two samples (1524) prior to further analysis. Microbiome alpha diversity (Chao1 richness and Faith’s phylogenetic diversity) and beta diversity (Bray–Curtis and Weighted UniFrac distances) were estimated with Qiime2.

### 2.5. Statistical analysis

All analyses were carried out in R v. 4.3.3 [42]. *DEseq2* [43] was used to compare ASV abundance between strains (DAN and HON9), diets (EDS and probiotic), environments (enriched and poor) and hatching mode (natural and artificial dechorionation). Low coverage ASVs were independently filtered, and the default settings were applied for outlier detection and moderation of ASV dispersion. ASV abundance was considered significantly different at false detection rate (FDR) < 0.05 and ASV relative abundance was visualized using *Pheatmap* [44], based on Euclidean distance clustering. To assess the effect of the incorporation of the probiotic in the diet on the microbiota composition of the gut, we also analysed the diet groups with the voom-limma approach implemented in *edgeR* [45], and compared the results with those from *DEseq2*. We used the *calcNormFactors* function to calculate the normalization factors. After normalization, *woom* from the package *limma* (version 3.28.14) [46] was used to transform the data for linear modelling by transforming the counts to log2-counts per million (logCPM) and compute observational-level weights based on the mean–variance relationship. We then used *lmFit* to fit linear models using weighted least squares for each taxa, to compare the differential abundance of taxa between the two diet groups based on Benjamini–Hochberg false FDR corrected *p*-values.

We analysed the influence of strain, diet, environment, size (weight, mg), hatching mode and their interactions on microbiome alpha diversity (Chao1 richness and Faith’s phylogenetic diversity) using generalized linear models. Model checks were carried out using the functions *check_model* and *check_normality* in the package *performance* [47]. A gamma distribution was used for Chao1 models and a Gaussian distribution in the case of Faith_pd. Model comparison was carried out using *glmulti* [48] and the best model was chosen based on the Akaike’s information corrected criterion (AICc) and likelihood-ratio chi-squared test (LRT). Given that models within 2 AIC units are equally plausible, we chose the simplest model with the fewest number of predictors [49]. All models within 2 AIC units are reported in electronic supplementary material, tables S4−S6.

Beta diversity was visualized with non-metric multidimensional scaling using *vegan* 2.6-4 [50] followed by multivariate analysis of variance (PERMANOVA) carried out using *adonis2* in *vegan*. Size, strain, environment, diet, hatching mode, activity and inspections were used as predictors and we computed 99 999 permutations to estimate the *p*-value of statistical significance.

Generalized linear models were also used to analyse the effects of size (weight), genotype (strain), environment (enriched/poor), diet (EDS/probiotic), hatching mode (natural/artificial dechorionation) and microbiome alpha diversity on behaviours (exploration, activity and inspections) and the best model was chosen using *glmulti* [48] based on AICc and LRT. Analyses using Chao1 and Faith_pd as predictors were carried out separately maintaining the rest of the predictive variables. A binomial distribution was used for exploration models (proportion of time) and negative binomial for activity and inspections (counts). We then used *MaAsLin2* (microbiome multivariable association with linear models) [51] to test for associations between individual bacteria (ASV) and behaviour (activity and inspections), with diet, hatching mode and strain as additional fixed factors. *MaAsLin2* uses linear mixed-effects models to test for statistically significant associations between individual taxa and the variables of interest, correcting for multiple tests with the Benjamini–Hochberg FDR method [51]. We used ASV abundance as input data, and the following parameters: analysis = ‘ZINB’, normalization = ‘NONE,’ transform = ‘NONE’, min_abundance = 1, min_prevalence = 0.15, min_significance = 0.05. With these parameters the minimum number samples represented per ASV was 7 (minimum *N* not zero).

## 3. Results

### 3.1. Microbiome composition and diversity

**Figure 1.**
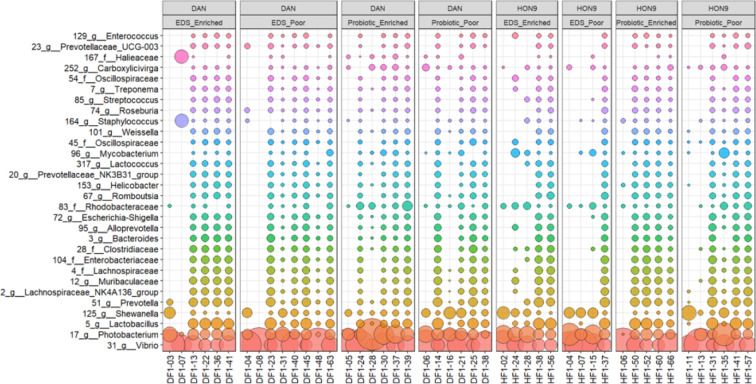
The most abundant 30 ASVs (family and genus level) observed across all samples (*n* = 46) of *Kryptolebias marmoratus*. DF and HF are individuals from each of the strains (DAN or HON9, respectively), EDS or probiotic indicate the different diet and poor or enriched indicate whether the fish were reared in standard (poor) tanks or those with environmental enrichment (plant and hide).

The most abundant ASVs corresponded to the phylum Proteobacteria, followed by Firmicutes and Bacteroidota. *Vibrio* was the most abundant genus throughout all samples (*n* = 46), followed by *Photobacterium*, *Lactobacillus*, *Shewanella* and *Prevotella* (figure 1). Analysing the distribution of ASVs between groups using *DEseq*2 identified 17, 38, 30 and 85 taxa that differed significantly in abundance between strains (DAN *n* = 26, HON9 *n* = 20), diets (EDS *n* = 23, probiotic *n* = 23), rearing environments (enriched *n* = 22, poor *n* = 24) and hatching modes (natural *n* = 21, artificial dechorionation *n* = 25), respectively (electronic supplementary material, table S2a−d). The analysis using *woom* identified seven ASVs at the family level differentially represented in fish fed with both diets, five of them also identified by *DEseq2* (Desulfovibrionaceae, Prevotellaceae, Rikenellaceae, Bacillaceae and Staphylococcaceae). Four more ASVs (*Tannerellaceae*, *Coriobacteriales_Incertae_Sedis*, *Mycobacteriaceae* and *Muribaculaceae*) that were identified as significant by *DEseq* and were significant in voom before FDR correction (electronic supplementary material, table S3). These results indicate a significant effect of the addition of the probiotic in the gut microbiome composition but there was no significant difference in alpha diversity between diets (Wilcoxon test: Chao1 *W* = 247, *p* = 0.711; Faith_pd *W* = 235, *p* = 0.527).

**Figure 2.**
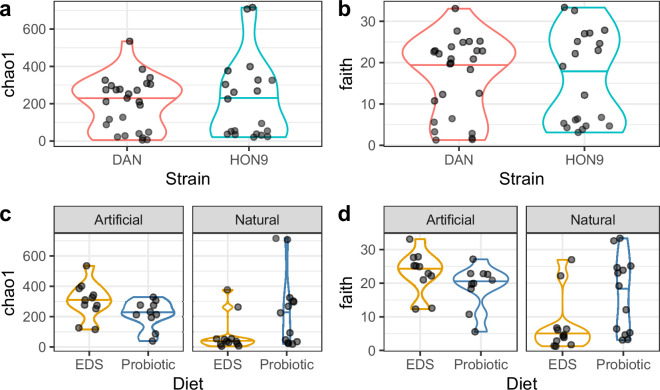
Influence of strain (DAN or HON9), hatching mode (natural or artificial dechorionation) and diet (standard EDS or probiotic enriched) on the alpha diversity of *Kryptobelias marmoratus*’ microbiota, measured as Chao1 and Faith’s phylogenetic diversity. The horizontal line in the violin plots denotes the median value.

**Figure 3.**
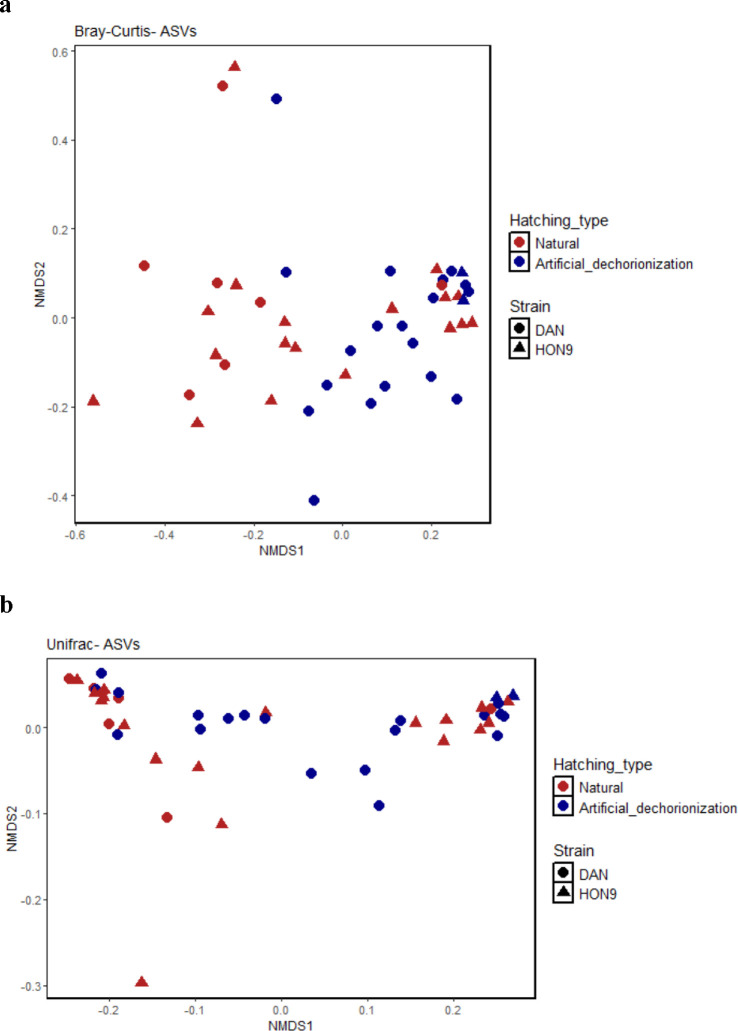
Influence of the hatching mode on beta diversity measures of gut microbiome. Non-metric multidimensional scaling (NMDS) ordination of the microbial gut community of *Kryptolebias marmoratus* strains (DAN, circles *n* = 26 and HON9, triangles *n* = 20) hatched naturally (red) or by artificial dechorionation (blue). (a) Bray–Curtis distance and (b) Weighted UniFrac distance.

All five models within 2 AIC units for Chao1 diversity (microbiome abundance and richness) included strain, diet and hatching type as predictors, with interactions among them. The simplest model of those within 2 AIC units for Chao1 diversity (microbiome abundance and richness) indicated that it was significantly influenced by strain (LR Chisq = 8.55, df = 1, *p* = 0.03; figure 2a), hatching mode (LR Chisq = 12.16, df = 1, *p* < 0.001) and the interaction between diet and hatching mode (LR Chisq = 8.44, df = 1, *p* = 0.004; figure 2c) (electronic supplementary material, tables S4 and S6, Model 3). The Faith’s phylogenetic diversity was also significantly influenced by strain (LR Chisq = 6.06, df = 1, *p* = 0.01; figure 2b), hatching mode (LR Chisq = 21.05, df = 1, *p* < 0.001), and the interaction between diet and hatching mode (LR Chisq = 7.05, df = 1, *p* = 0.008; figure 2d) (electronic supplementary material, tables S4 and S6, Model 1). Fish from the DAN strain and fish artificially hatched had higher alpha diversity (both Chao1 and Faith_pd) than those from HON9 and naturally hatched, respectively, and fish fed with probiotics displayed higher diversity in fish naturally hatched but the opposite trend in those artificially dechorionated. By contrast with alpha diversity, only hatching mode influenced beta diversity metrics (Bray–Curtis distance, df = 1, *F* = 5.15, *p* < 0.001 and Weighted Unifrac distance, df = 1, *F* = 6.68, *p* = 0.01) (electronic supplementary material, table S5; figure 3).

### 3.2. Association between fish behaviour and microbiome

**Figure 4.**
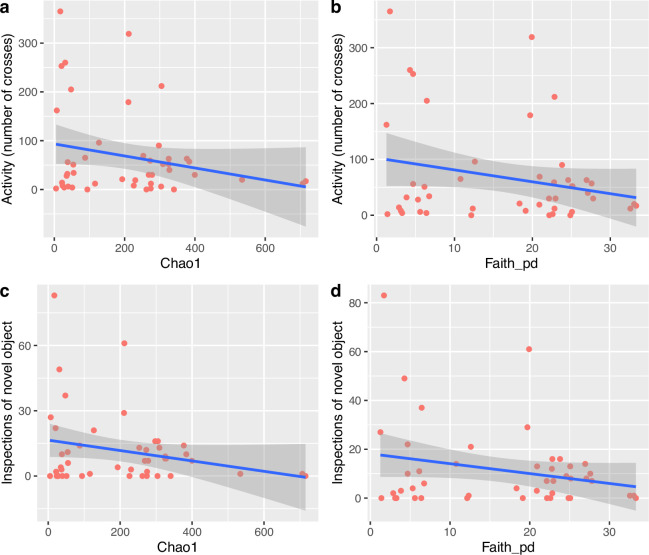
Relationship between behaviour (activity and inspections measured using a novel object test) of *K. marmoratus* (*n* = 46) and alpha diversity (Chao1 and Faith_pd).

**Figure 5.**
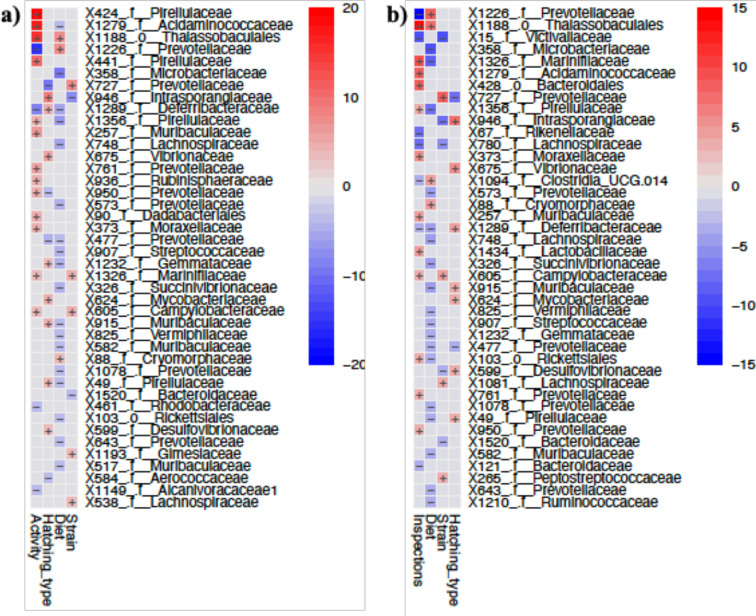
Significant associations (Padj after Benjamini–Hochberg correction for multiple testing = 0.05) between individual ASVs and behaviour, strain (baseline value = HON9), diet (baseline value = probiotic) and hatching mode (baseline value = natural) estimated using *MaAsLin2*. (a) Models including activity and (b) models including inspections.

The best models for exploration time did not include any of the predictors, indicating that neither microbiome alpha diversity (Chao1 or Faith_pd) nor fish size, rearing environment, diet or hatching mode significantly influenced this behaviour (electronic supplementary material, tables S5 and S6). By contrast, the best models for activity included alpha diversity, but none of the other factors, as significant predictors when using Chao1 as predictor (Model 1 Strain: LR = 2.51, df = 1, *p* = 0.11; Chao1: LR = 5.27, df = 1, *p* = 0.02) and with Faith_pd as predictor (Model 1 Strain: LR = 3.10, df = 1, *p* = 0.08; Faith: LR = 4.74, df = 1, *p* = 0.03). Similar results were obtained for inspections, where the best models included alpha diversity as significant predictors, both Chao1 (Model 1 Strain: LR = 3.37, df = 1, *p* = 0.07; Chao1: LR = 6.76, df = 1, *p* = 0.01 and Strain: LR = 3.82, df = 1, *p* = 0.05) and Faith_Pd (Model 1 Strain: LR = 3.83, df = 1, *p* = 0.05; Faith: LR = 4.83, df = 1, *p* = 0.03), but none of the other factors (electronic supplementary material, tables S5 and S6; figure 4). Given that alpha diversity was a significant predictor of activity and inspections, we then ran multivariate association analyses, to identify individual ASVs potentially related to the fishes’ behaviour. Using *MaAsLin2*, and based on adjusted probabilities, we identified 44 ASVs significantly associated with activity, strain, hatching mode and/or diet. Seventeen of those were significantly associated with activity; nine were only associated with activity, four were associated with activity and diet, two with activity and strain, one with activity and hatching mode and one was associated with the four factors (electronic supplementary material, table S7; figure 5). Regarding fish inspections, 42 ASVs were significantly associated with strain, hatching mode and/or diet. Nineteen of these were significantly associated with inspections; ten were associated with inspections only, six with inspections and diet, two with inspections and strain, and one with inspections, diet and hatching mode (electronic supplementary material, table S8; figure 5). Eleven ASVs were significantly associated with both activity and inspections (table 1).

**Table 1.** Results from microbiome multivariable association analyses carried output using *MAasLin2*, showing the correlation coefficient (coef) of the individual bacterial taxa and fish behaviour (activity and inspections), standard error (stderr) and statistical significance (*q*-value with Benjamini–Hochberg correction for multiple testing). Only taxa common to both behaviours are represented.

						activity			inspections		
bacterial feature (ASV)	phylum	class	order	family	genus	coef	stderr	*q*-value	coef	stderr	*q*-value
Thalassobaculales	Proteobacteria	Alphaproteobacteria	Thalassobaculales	uncultured	uncultured	0.732	0.119	2.550 × 10^−7^	0.708	0.115	5.880 × 10^−7^
Prevotellaceae	Bacteroidota	Bacteroidia	Bacteroidales	Prevotellaceae	*Prevotellaceae_UCG-003*	−2.523	0.408	2.550 × 10^−7^	−2.353	0.376	5.467 × 10^−7^
Acidaminococcaceae	Firmicutes	Negativicutes	Acidaminococcales	Acidaminococcaceae	*Phascolarctobacterium*	2.492	0.377	2.860 × 10^−8^	2.079	0.424	1.918 × 10^−4^
Deferribacteraceae	Deferribacterota	Deferribacteres	Deferribacterales	Deferribacteraceae	*Mucispirillum*	−3.166	0.658	2.374 × 10^−4^	−1.848	0.548	2.494 × 10^−2^
Marinifilaceae	Bacteroidota	Bacteroidia	Bacteroidales	Marinifilaceae	*Odoribacter*	0.887	0.284	4.594 × 10^−2^	2.832	0.561	1.067 × 10^−4^
Pirellulaceae	Planctomycetota	Planctomycetes	Pirellulales	Pirellulaceae	*Pirellula*	0.800	0.217	1.154 × 10^−2^	0.564	0.168	2.499 × 10^−2^
Muribaculaceae	Bacteroidota	Bacteroidia	Bacteroidales	Muribaculaceae	*Muribaculaceae*	2.712	0.619	1.126 × 10^−3^	1.655	0.447	1.246 × 10^−2^
Moraxellaceae	Proteobacteria	Gammaproteobacteria	Pseudomonadales	Moraxellaceae	*Acinetobacter*	0.439	0.119	1.154 × 10^−2^	0.432	0.106	3.366 × 10^−3^
Campylobacteraceae	Campilobacterota	Campylobacteria	Campylobacterales	Campylobacteraceae	*Campylobacter*	0.599	0.181	3.231 × 10^−2^	0.547	0.171	3.917 × 10^−2^
Prevotellaceae	Bacteroidota	Bacteroidia	Bacteroidales	Prevotellaceae	*Alloprevotella*	1.386	0.351	6.039 × 10^−3^	1.348	0.398	2.377× 10^−2^
Prevotellaceae	Bacteroidota	Bacteroidia	Bacteroidales	Prevotellaceae	*Alloprevotella*	0.745	0.194	8.031 × 10^−3^	0.762	0.231	2.926 × 10^−2^

## 4. Discussion

The effect of the gut microbiome on the mammalian brain is well established [52] and mounting evidence suggests that a disruption of the ‘microbiota–gut–brain axis’ is associated with behavioural and cognitive diseases in humans [53]. However, the link between microbiome and behaviour in animals, apart from models like mice [54] or more recently zebrafish, remains largely unexplored [55]. Here, by manipulating the rearing environment and diet, and assessing fish from different genotypes, we identified a strong association between fish gut microbiota (composition and diversity) and behaviour (activity level and inspections of a novel object), but no significant influence of strain, diet, environment or hatching mode on behaviour. However, we found that the microbiome composition and diversity were significantly influenced by strain, hatching mode and, to a lesser extent, diet. We also found that the particular taxa influenced by these factors were not necessarily the same associated with the behaviour, suggesting an indirect effect of the host genetics and environmental conditions on fish behaviour, through the modification of the ecological conditions of their gut microbiota [56]. This supports the theory that behavioural effects of the microbiome may be a byproduct of the local effects that the microbial compounds exert on the host physiology, or as a side effect of the metabolites that the microbiota need to grow in the gut environment, of which the host can become dependent [57].

Our results indicate that genetic background, hatching mode and the interaction between hatching mode and diet shaped the fish gut microbiome composition and diversity. Although the evidence for phylosymbiosis (higher intraspecific than interspecific similarity in the structuring of the microbial communities) in fish is generally weak [58,59], individual genotypes can influence the gut microbiota composition [60], which may explain the difference we observed between strains. In addition, a small proportion of the gut microbial community present before first feeding can be inherited from the egg, potentially via transovarial maternal contribution [61], and remain during development [62]. This could explain the differences we found between naturally hatched and artificially dechorionated embryos, if the artificial hatching disrupted the microbiota transmission from the egg. The egg microbiome is acquired through oviposition and is immunologically important for the larvae, for example to prevent fungal infections [63]. Given that the egg microbiome provides most of the initial colonizers to which the immune system is exposed during early development, we suggest that artificial dechorionation may affect the fish immune response later in life. The initial microbiome composition is also important for neurodevelopment; for example, early colonization of the zebrafish gut by *Vibrio cholerae* and *Aeromonas veronii* seems to be critical for its normal neurobehavioural development [12]. In catfish, the genera present from the egg stage include *Bacillus* and *Enterococcus*, both potentially probiotic, and *Tenacibaculum* and *Vibrio*, potentially pathogenic [62]. *Vibrio* was one of the genera which significantly differed between naturally and artificially hatched fish here, with higher abundance in the artificially dechorionated fish. Other taxa, such as *Photobacterium*, also common in the study fish, seem to colonize the gut later during the development. Both *Vibrio* and *Photobacterium* are common in aquaculture systems and can be acquired through live feeds (rotifers) [64], explaining their high prevalence and the observed interaction between hatching mode and diet in shaping microbiome diversity. By contrast, the physical enrichment of the rearing environment did not influence the microbiome diversity or community structure.

Despite the effect of the strain, hatching mode and diet on the microbiome composition and diversity, none of these factors seemed to play a role on fish behaviour, measured as activity and number of inspections of a novel object (neophobia). However, microbiome alpha diversity (both species and phylogenetic diversity), as well as specific taxa, significantly influenced behaviour. Alpha diversity was negatively correlated with both behaviours; fish with higher Chao1 and Faith_pd diversities displayed lower levels of activity and inspection. These results mirror the decline in activity with increasing Faith_pd skin diversity previously observed in female guppies [14]. Dysbiosis due to infection, hormonal production or olfactory regulation were proposed to explain the correlation in the case of the guppies, but whether there is a causal relationship and its possible direction was unclear [14]. Understanding the direction of the microbiome–behaviour relationship is particularly challenging, as the host genetics influences gut microbiota composition, which in turn affects immunity and brain development, through differences in released metabolites [57]. The advantages of using an animal model like ours, which allows one to separate genetic from environmental influences, are evident.

We found that the incorporation of a probiotic resulted in an increase of the taxa from the order Bacteroidales (class Bacteriodota) (four families), as well as representatives from Coriobacteriales, Corynebacteriales, Desulfovibrionales, Bacillales and Staphylococcales. In fish, Bacillales tend to appear in microbiomes from plant-based diets, with decreased microbial diversity, while Bacteriodales are more common in relation to the presence of animal protein in the diet [65]. Bacteriodota are correlated with high production of short chain fatty acids (SCFAs), which contribute to both the host nutrition and signalling pathways [66]. The associations between behaviour (both activity and inspections) and individual taxa represented the main proportion of those identified by the multiple association analysis (39% and 45%, respectively). Five of the 11 individual ASVs which influenced both activity and inspections were Bacteriodales, which represented the most common order associated to the behaviours studied. The order Bacteriodales has been related to neurological disorders in humans [67] and was found in low abundance in children with autism spectrum disorder, potentially because of metabolite dysregulation affecting the intestinal mucosal cells and consequently the host immune response and neurological function [68]. Three ASVs, *Thalassobaculales*, *Prevotellaceae* and *Pirellulaceae*, were associated with changes both in diet (positively in the first two cases and negatively in the third) and behaviour (activity and inspections; negatively in the case of *Prevotellaceae*, positive in the other two cases). *Prevotellaceae* is known to influence health in humans and spatial memory in mice, under conditions of high fat diet and low oestrogen [69]. By contrast, none of the ASVs were simultaneously associated with both behaviours and the remaining factors (strain, diet or hatching mode). Thus, it could be that the inclusion of the probiotic in the diet influenced the gut microbiota of the study fish, directly or indirectly altering the abundance of taxa associated to neurological development, which could explain the observed relationship between the alpha diversity and individual taxa, and the killifish behaviour.

## 5. Conclusions

We found that host genetics (strain), hatching mode (potentially influencing early gut colonization) and the inclusion of a probiotic in the diet, but not environmental enrichment, affected the gut microbiota composition of the killifish, increasing the abundance of Bacteroidales, which in mammals are related to the production of SCFAs and neurodevelopment. We also found that a high microbiota diversity and the presence of specific taxa were associated with increased activity and inspections of a novel object, which are behaviours typically used to assess fear and anxiety-like behaviours while the particular ASVs influenced by genetics, hatching and diet were not the same as those associated with differences behaviour, the highest proportion of them belonged to the order Bacteriodales in both cases. We suggest that the observed association between microbiome and fish behaviour could be an indirect effect of the modulation of the ecological conditions of the gut microbiota by the host genetics and rearing conditions (hatching and diet), which could be affecting the production of microbial metabolites that interact with the fish physiology.

## Data Availability

Sequence data that support the findings of this study have been deposited in the European Nucleotide Archive (ENA) under accession number PRJEB76690. Supplementary material is available online [70].
